# Noise-induced hearing loss alters potassium-chloride cotransporter KCC2 and GABA inhibition in the auditory centers

**DOI:** 10.1038/s41598-024-60858-1

**Published:** 2024-05-09

**Authors:** V. Parameshwarappa, M. I. Siponen, I. Watabe, A. Karkaba, A. Galazyuk, A. J. Noreña

**Affiliations:** 1 Laboratory of Cognitive Neurosciences, Centre National de la Recherche Scientifique, Aix-Marseille University, 3 Place Victor Hugo, 13003 Marseille, France; 2 Department of Anatomy and Neurobiology, Northeast Ohio Medical University, Rootstown, OH USA

**Keywords:** Midbrain, Auditory system, Sensory processing

## Abstract

Homeostatic plasticity, the ability of neurons to maintain their averaged activity constant around a set point value, is thought to account for the central hyperactivity after hearing loss. Here, we investigated the putative role of GABAergic neurotransmission in this mechanism after a noise-induced hearing loss larger than 50 dB in high frequencies in guinea pigs. The effect of GABAergic inhibition is linked to the normal functioning of K + –Cl– co-transporter isoform 2 (KCC2) which maintains a low intracellular concentration of chloride. The expression of membrane KCC2 were investigated before and after noise trauma in the ventral and dorsal cochlear nucleus (VCN and DCN, respectively) and in the inferior colliculus (IC). Moreover, the effect of gabazine (GBZ), a GABA antagonist, was also studied on the neural activity in IC. We show that KCC2 is downregulated in VCN, DCN and IC 3 days after noise trauma, and in DCN and IC 30 days after the trauma. As expected, GBZ application in the IC of control animals resulted in an increase of spontaneous and stimulus-evoked activity. In the noise exposed animals, on the other hand, GBZ application decreased the stimulus-evoked activity in IC neurons. The functional implications of these central changes are discussed.

## Introduction

It is well known that partial or complete sensory deafferentation is followed by dramatic central changes throughout the sensory centers^1,2^. In the auditory system, noise trauma is often used to produce partial cochlear hearing loss in animals. The level of cochlear damages depends on the level and exposure duration of the noise trauma^3^. The effects of noise-induced hearing loss have been well documented at many levels of the central auditory system (from the cochlear nucleus to auditory cortex), in several animal species (rats, guinea pigs, gerbils, mice), and in both anesthetized and awake preparations. In brief, noise-induced hearing loss is followed by systematic increase of stimulus-evoked and spontaneous neural activity, both immediately and weeks after noise trauma^2,4–7^.

These widespread central changes after sensory deafferentation have been interpreted as result of homeostatic plasticity^8,9^. Homeostatic plasticity regroups a set of adaptive mechanisms to preserve mean neural activity around a set point value. Homeostatic plasticity may also play a role in maintaining neural coding efficiency when the distribution of sensory inputs (mean and variance) is changing^7,10–13^. The repertoire of the molecular mechanisms participating to homeostatic plasticity is likely immense^8,14–19^. Among these mechanisms, the inhibitory neurotransmission plays a central role in adjusting neural activity in the central sensory systems due to partial deafferentation^4,20–22^. A downregulation of GABAergic and glycinergic inhibition throughout the auditory central nervous system has been reported as an effect of aging, noise trauma or cochleoctomy^4,20,23,24^. The inhibitory effect of GABA and glycine depends on the low intra-cellular concentration of chloride ions (relative to the extra-cellular compartment), that is maintained low by K + ‒Cl- co-transporter isoform 2 (KCC2) located in the neuron’s plasmatic membrane. To note, Na + -K + -Cl- co-transporter (NKCC1) is a membrane protein that transport sodium, potassium and chloride into neurons. Importantly, KCC2 is downregulated during the early phase of development. This is the reverse for NKCC1: it is expressed during development but not in adulthood. As a consequence, GABA has been found to be excitatory during development^25^. Interestingly, in a model of peripheral neuropathy induced by chronically constricting the sciatic nerve in adults rats, it has been shown that KCC2 is down-regulated and that GABA induces excitatory synaptic current^26^. These studies suggest that the mechanisms of GABAergic homeostatic plasticity involve not only the secretion of GABA or the GABA receptors, but also the KCC2, which eventually “decides” if GABA is inhibitory or excitatory.

In auditory modality, very few studies have investigated the effects of sensory deafferentation on KCC2 and chloride homeostasis^27,28^. After bilateral cochlear ablation, no change in the expression of KCC2 mRNA or protein in IC was reported^27^. However, the results of this latter study suggest that profound hearing loss leads to the disruption of chloride homeostasis. In a recent study carried out in our laboratory, we assessed the effects of unilateral cochlear nerve transection on the KCC2 located in the plasmatic membrane of neurons in the cochlear nucleus^28^. We showed a strong downregulation of KCC2 in the cochlear nucleus ipsilateral to the cochlear damage, at 3 and 30 days after the cochlear nerve resection. This result is consistent with those obtained in other sensory systems^21,29^.

The present study is aimed at investigating further the mechanisms of GABAergic homeostatic plasticity after noise-induced hearing loss. The first goal of the study was to investigate if a partial hearing loss, induced by noise trauma, can alter the expression of membrane KCC2 (and NKCC1) in the VCN, DCN and IC. The second goal was to assess the effects of Gabazine, a GABA antagonist, on neural activity in IC. The IC was chosen for electrophysiology because it is a “sensory crossroads” and the main output of the VCN and DCN. Furthermore, as it is located higher up in the central auditory system than the VCN and DCN, we felt that neural changes in the IC would be more relevant to the effects of noise trauma on auditory perception (tinnitus, hyperacusis).In control animals, Gabazine is expected to increase the spontaneous and stimulus-evoked neural activity. In exposed animals, on the other hand, if KCC2 is downregulated after noise-induced hearing loss, Gabazine may have a reduced excitatory effect, or even an inhibitory effect.

## Results

**Figure 1:**
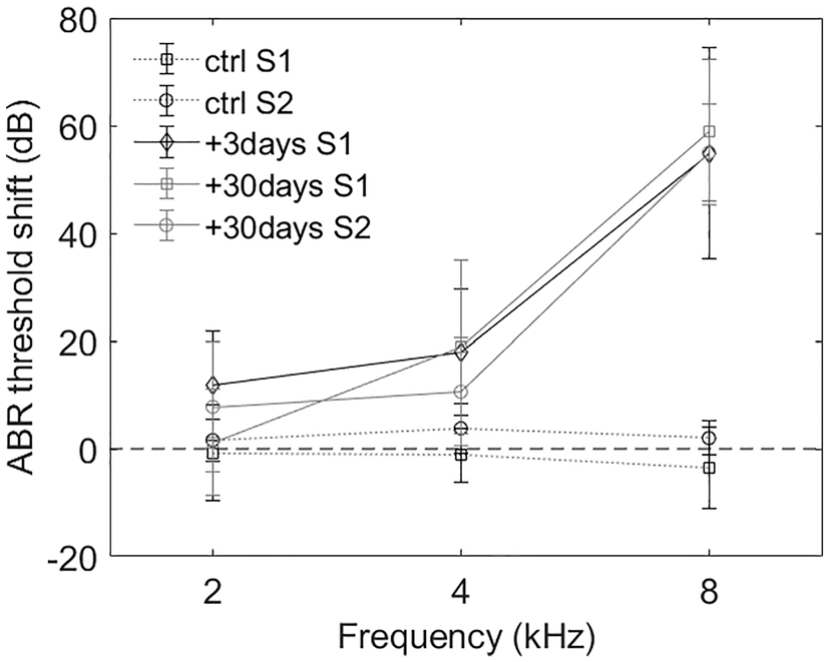
Averaged ABR threshold shift in all animals of the two studies (S1: immunohistochemical study—n = 12 animals, S2: electrophysiological study, n = 16 animals).

ABRs of all animals were tested to assess their hearing thresholds and the noise-induced hearing loss (Fig. 1). Consistent with our previous study^30^, the noise-induced hearing loss at 3 days and 30 days after the noise trauma resulted in more than 50 dB ABR threshold shift at high frequencies (8 kHz and above).

### Immunochemistry: effects of noise-induced hearing on KCC2

**Figure 2:**
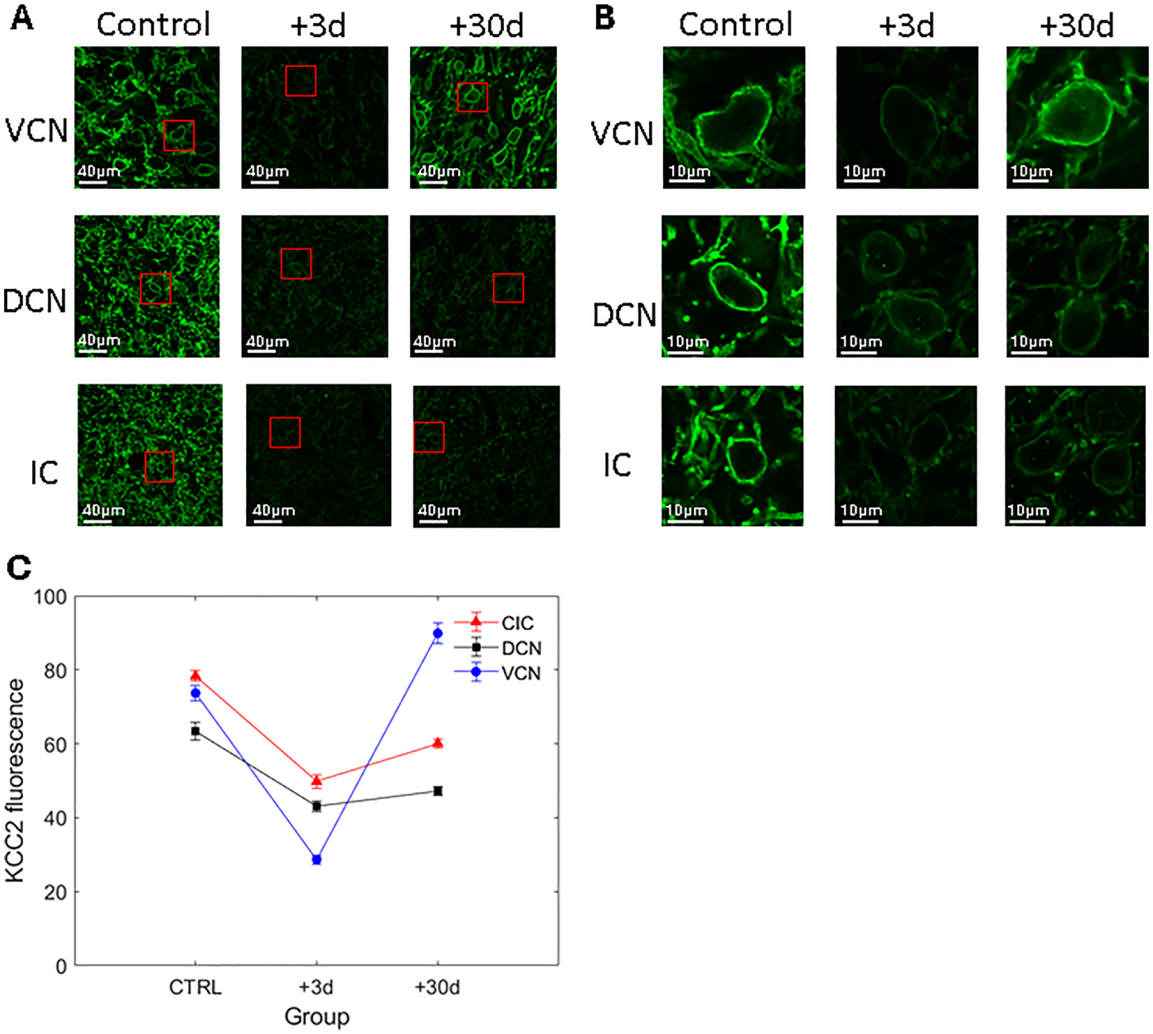
(**A**) Examples of slices showing KCC2-related fluorescence in control (first column) and exposed animals (second and third columns: + 3d and + 30d after noise trauma, respectively) in the VCN (first row), DCN (second row) and IC (third row). The region of interests delineated by red rectangles are shown in B at higher magnification. (**B**) Zoom on selected cell bodies. (**C**) Averaged KCC2-related fluorescence in control (n = 4 animals) and exposed animals (+ 3d: n = 4 animals, + 30d: n = 4 animals) in VCN, DCN, and IC.

Examples of entire slices showing KCC2-related fluorescence in control and exposed animals are shown in Fig. 2.

Simple visual inspection of examples shown in Fig. 2A,B suggests that KCC2 expression is dramatically downregulated in the VCN, DCN and IC at 3 days post-trauma, and in the DCN and IC at 30 days post-trauma. The averaged quantified immunofluorescence in the different groups is shown in Fig. 2C. The analysis of variance indicated that there is a significant interaction between the time and the central region (F(4,1617) = 76,16, *p* < 0.001). The Tuckey test revealed that the immunofluorescence is reduced in VCN, DCN and IC 3 days after the trauma compared to the control group (*p* < 0.001). Between + 3 days and + 30 days post-trauma, the immunofluorescence was unchanged in the DCN (*p* > 0.05), but was enhanced in the VCN and IC (*p* < 0.01). Finally, between control and + 30 days post-trauma, the immunofluorescence was reduced in the DCN and IC, and enhanced in the VCN (*p* < 0.001).

We also studied NKC1 expression in control and exposed animals. We observed no immunofluorescence in any of the central nuclei studied (VCN, DCN and IC). On a few sections, however, it was possible to visualize the cochlear nerve, and interestingly, we could observe that the cochlear nerve fluoresced with the NKCC1 antibodies (supplementary Fig. 1). These results demonstrates that the antibodies used in this study to reveal membrane NKCC1 were functioning properly and thus that NKCC1 is not expressed in the central auditory system, at least VCN, DCN and IC, of adult guinea pigs.

### Electrophysiology: effects of GBZ on neural activity

While the immunochemistry study revealed that KCC2 was globally downregulated in DCN, VCN and IC 3 days after noise trauma, and in DCN and IC 30 days after the trauma, the functional effect of this downregulation on neural activity is unknown. To assess the effect of KCC2 downregulation on GABAergic inhibition, the neural activity in IC was investigated before and after the application of a GABA antagonist Gabazine (GBZ). We expect GBZ to be excitatory in control animals, whereas less excitatory or even inhibitory in exposed animals. The data presented in this section are derived from 97 recordings performed in the control group (8 animals) and 85 recordings in the exposed group (8 animals).

First, to test for the stability of our recordings, neural data were collected during 45 min before GBZ administration. Tuning curves, STRFs and spontaneous activity were obtained twice (supplementary Fig. 2). We did not find any significant changes in evoked (TCs and STRFs) or spontaneous neural activity either in control or in exposed animals (Wilcoxon rank-sum test, p > 0.05, supplementary Fig. 2). The recordings used as baseline data before GBZ administration were those from the recordings just before GBZ application (“before 2” in supplementary Fig. 2).

**Figure 3:**
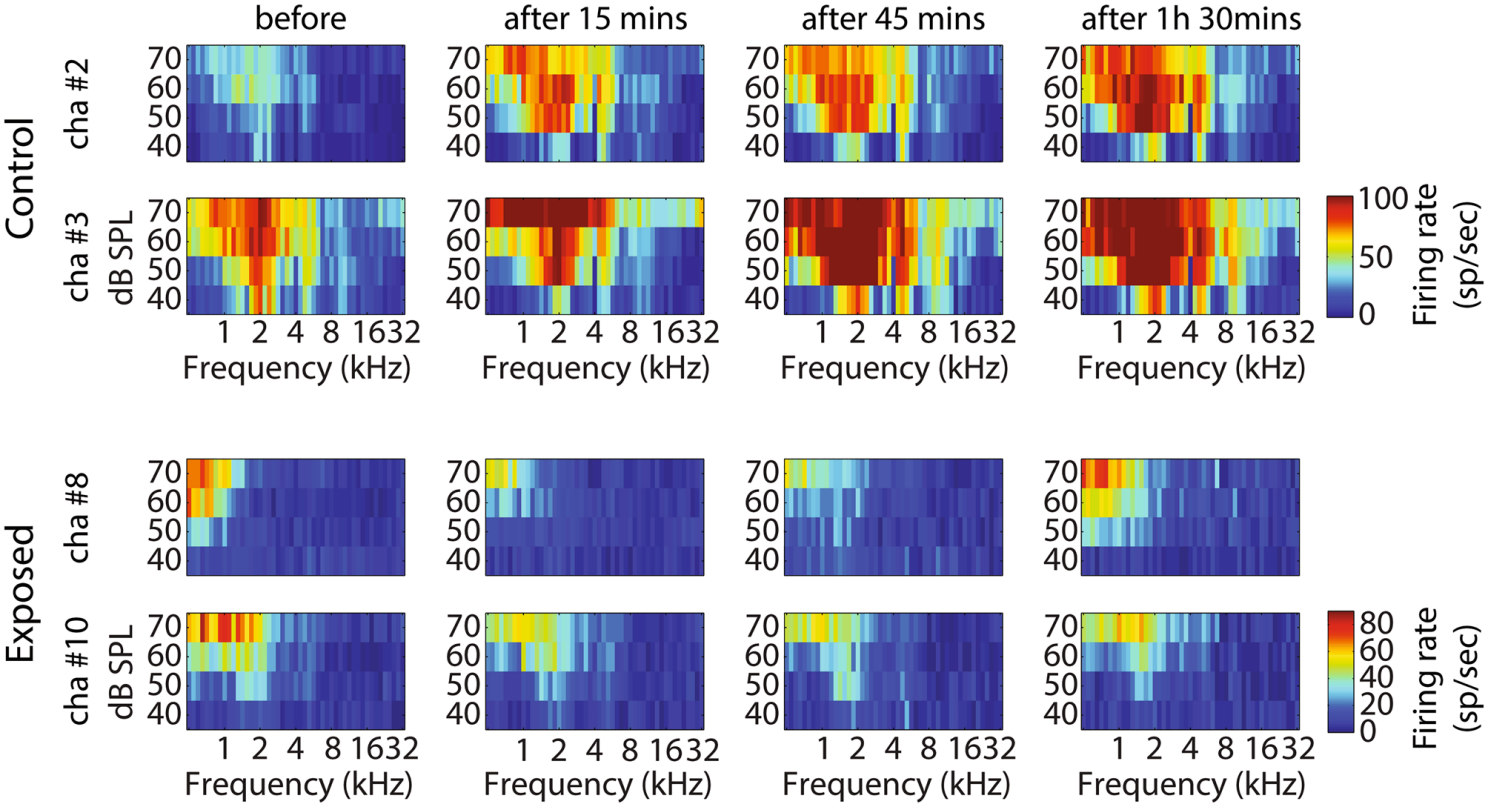
Example tuning curves (TCs) obtained in two animals (first and second rows: control, third and fourth rows: noise exposed) from two electrodes, before and at different time points after GBZ application. The firing rate of stimulus-evoked responses is coded by a colour code (from blue to red, i.e. representing from low to higher firing rate, see colour bar on the right).

**Figure 4:**
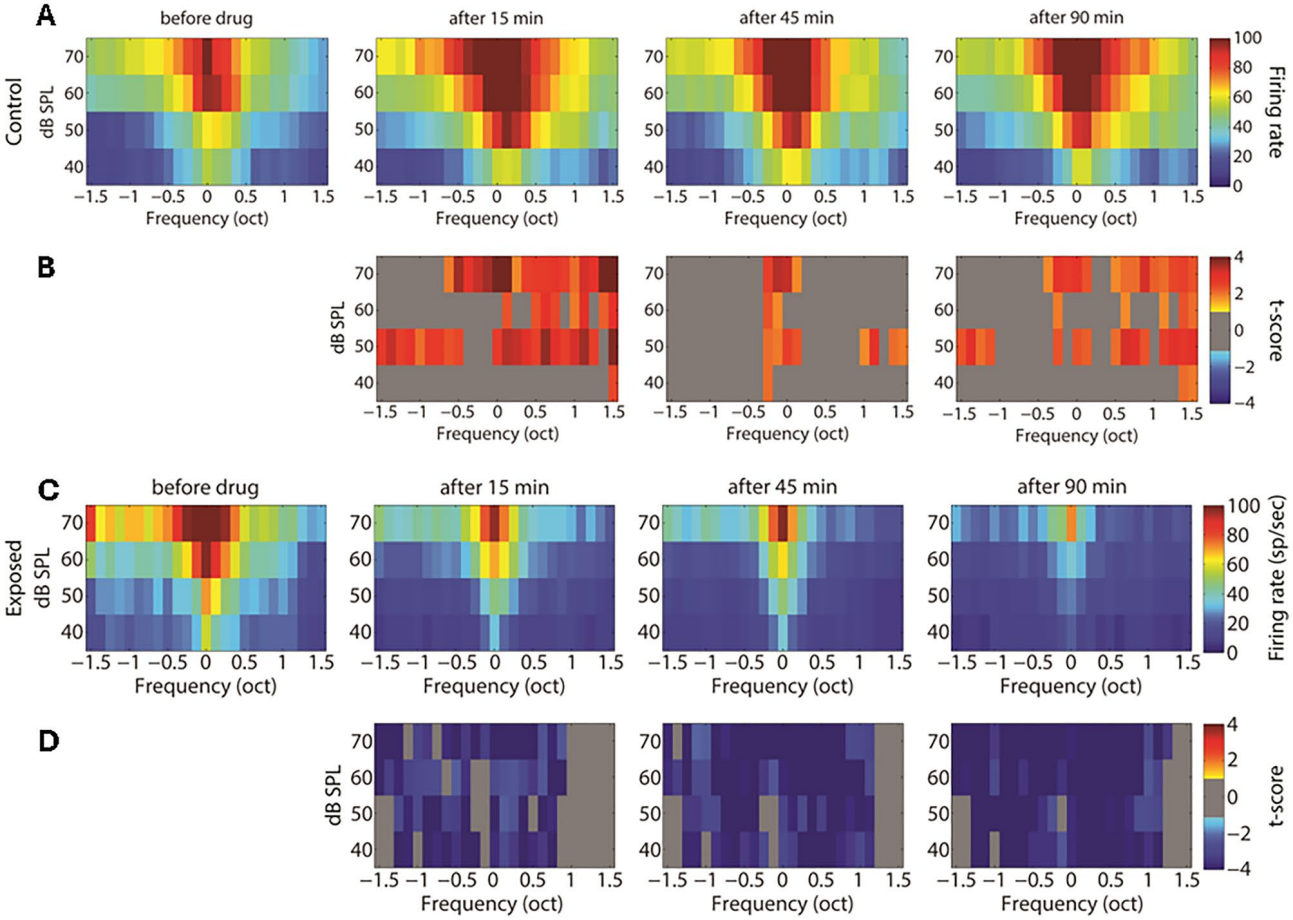
Averaged tuning curves over all electrodes and animals in the control (**A**, n = 8 animals) and exposed (**C**, n = 8 animals) groups before and after GBZ application and the corresponding cluster statistics (**B** and **D**, respectively).

GBZ application affected neuronal activity in the control and sound exposed groups, but in opposite directions. Examples of this effect on tuning curves are shown in Fig. 3. The average tuning curves before and after GBZ application in control and noise exposed groups are shown in Fig. 4. For averaging, the tuning curves were all aligned according to their characteristic frequency (CF) (frequency was then expressed as octave difference from CF). In control group, application of GBZ induced two effects on tuning curves at all measured time points (Fig. 4): (1) the spiking activity was increased at many frequency-level tone pip combinations, and (2) some responses were “unmasked” (new responses were evident at some frequency-level tone pip combinations). The application of GBZ in the noise exposed group had the opposite effects (Fig. 4): (1) the spiking activity was globally reduced at many, if not all, frequency-level tone pip combinations, and (2) some responses were even suppressed at some combinations.

The effect of GBZ was also studied on STRFs, which were obtained in response to multi-tone pip stimuli. The main reason for using such stimuli was to test the effect of GBZ with more complex stimulus paradigm compared to just isolated tone pips where a complex (more ecological) balance between excitation and inhibition take place.

**Figure 5:**
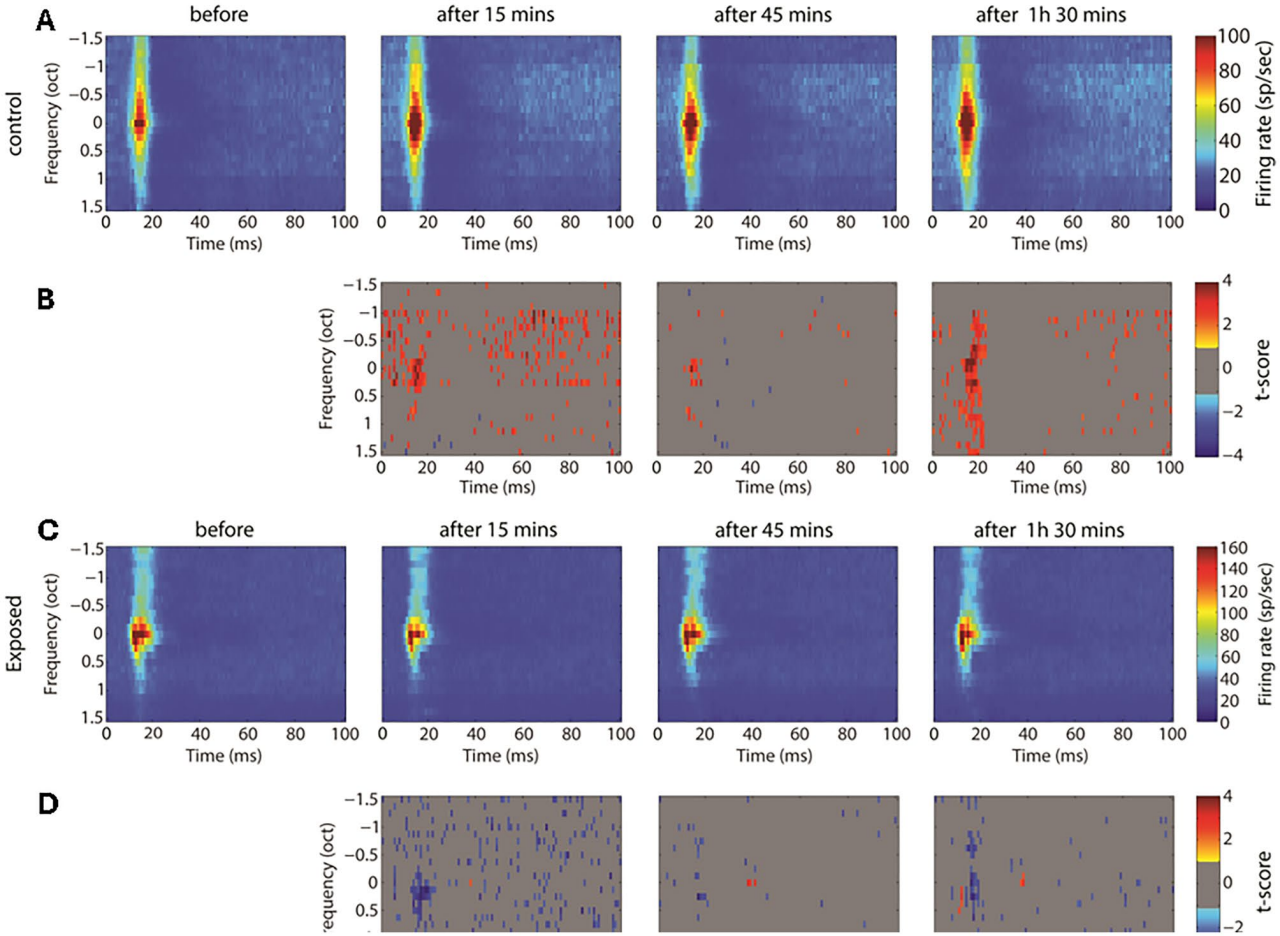
Averaged STRFs over all electrodes and animals in the control (**A**, n = 8 animals) and exposed (**C**, n = 8 animals) groups before and after GBZ application and the corresponding cluster statistics (**B** and **D**).

Figure 5 shows the averaged STRFs obtained from MUA in the control and exposed groups, respectively. For averaging, STRFs were all aligned according to their BF (frequency is then expressed as octave difference from BF). In the control group, GBZ application increased the evoked spiking activity at all measured time points, mainly at around BF, but also at other frequencies (*p* < 0.05). In contrast to the control group, spiking activity decreased after GBZ application in the noise exposed group, mainly around BF (*p* < 0.05).

**Figure 6:**
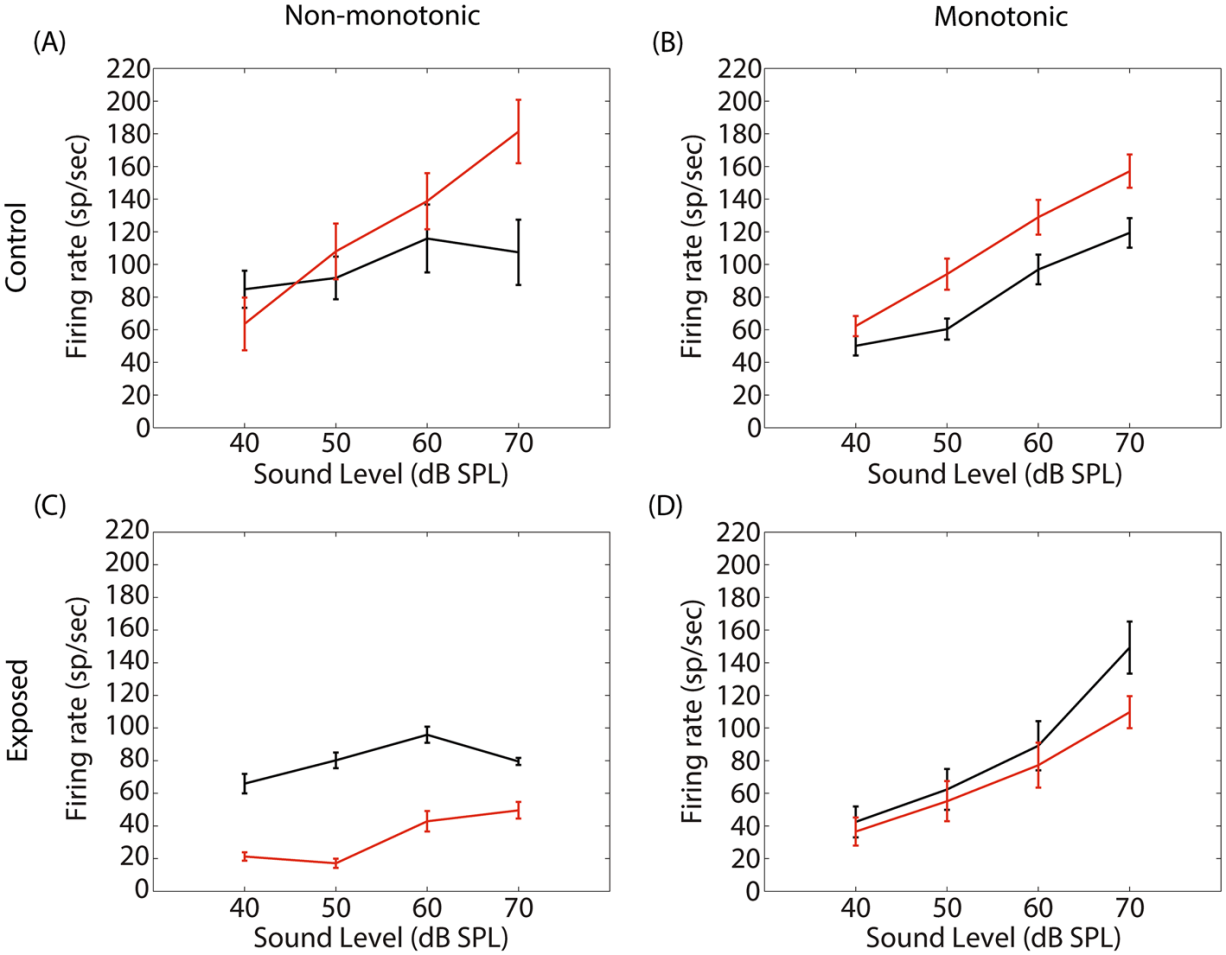
Averaged stimulus-evoked MUA at BF before (black line) and after GBZ application (red line) in control (first row) and noise-exposed (second row) groups. The recordings have been further categorized as non-monotonic (left column) or monotonic (right column).

Figure 6 shows the firing rate at BF as a function of sound level in the control and exposed animals before and 15 min after GBZ application.

The firing rate (expressed as sp/sec) was derived from the time window 10–50 ms post stimulus onset (the number of spikes is divided by the number of repetitions and then multiplied by 25). The recordings have been divided into two groups: multi-units (before GBZ application) with maximum firing rate at 70 dB were labelled as “monotonic”, or as “non-monotonic” otherwise. In the control group, the number of monotonic (Fig. 6B) and non-monotonic (Fig. 6A) units were 55 and 42, respectively. In the exposed group, the number of monotonic (Fig. 6D) and non-monotonic (Fig. 6C) units were 56 and 29, respectively. The Fisher exact test revealed that the percentage of monotonic and non-monotonic units were not significantly different between the control and exposed groups (*p* > 0.05). A 3 factors (rate-level function, time and sound level) linear mixed-effects analysis (see methods) was used to evaluate the statistical changes caused by GBZ application in each group (control and exposed). In the control group, the sound level factor was significant (F = 59.16, *p* < 0.001) as well as the two-way interaction between rate-level function and time (F = 4.239, *p* < 0.05) and the tree-way interaction between all factors (F = 10.288, *p* < 0.05). Post-hoc analysis revealed that GBZ had a significant effect at 70 dB in the non-monotonic units. This result is consistent with earlier studies which have shown that GBZ changed non-monotonic units into monotonic ones^31^. In the exposed group, all factors taken individually were significant (sound level: F = 203.59, *p* < 0.001, time: F = 32.72, *p* < 0.001, rate-level function: F = 23.137, *p* < 0.001). Moreover, the two-way interaction between rate-level function and time (F = 10.99, *p* < 0.01) and the three-way interaction between all factors were also significant (F = 18.57, *p* < 0.001). Post-hoc analysis revealed that GBZ had a significant effect in non-monotonic units from 40 to 60 dB.

**Figure 7:**
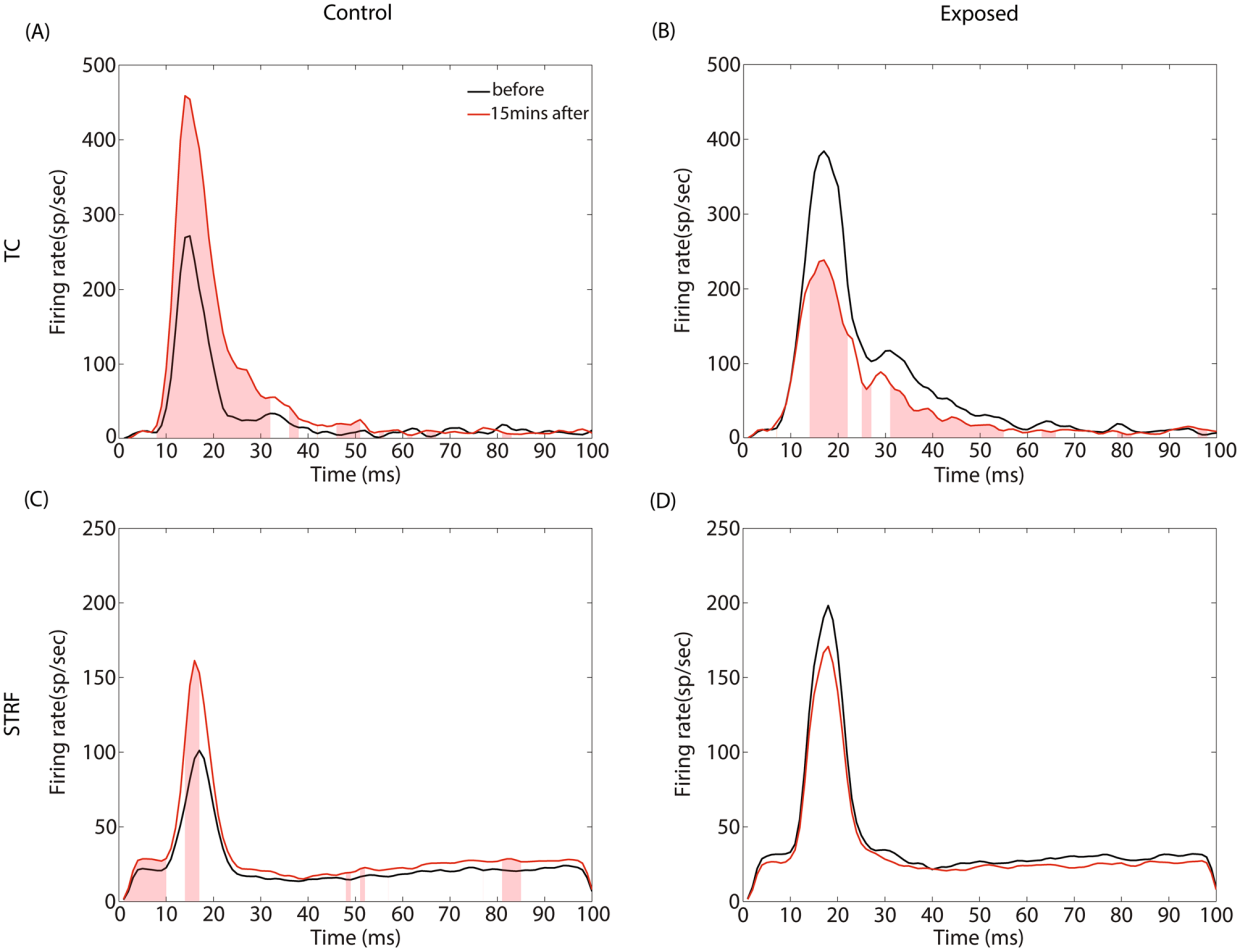
Averaged peri-stimulus time histograms at BF before (black line) and after GBZ application (red line) in control (first column) and noise-exposed (second column) groups for the two stimulus conditions (TC: first row, and STRF: second row). Red shaded area indicates the time points where GBZ significantly changes the FR.

The effects of GBZ on the PSTH for TC and STRF are shown in Fig. 7. The firing rate in PSTHs has been derived at BF and 70 dB, and are expressed in spikes/sec (sp/sec) (the number of spikes in each bin is divided by the number of repetitions and then multiplied by 1000). Changes in PSTH were statistically tested at each time bin using a non-parametric cluster test (*see* Methods) and the significant changes 15 min after GBX application compared to before are displayed as red shade area. For STRF-controls, GBZ is associated to an increase of the peak response, but it does not change the general shape of PSTH. For TC-controls, GBZ increases the peak response and the response duration. For STRF-exposed group, GBZ does not change significantly the PSTH. On the other hand, for TC-exposed group, GBZ decreases the peak response and the duration of the response, which is the opposite pattern of that observed in the controls. One notes that the averaged peak firing rate for TC-controls (~ 270 spikes/sec) is similar to the peak firing rate for TC-exposed animals after GBZ application (~ 230 spikes/sec). Interestingly, the averaged peak firing rate for TC-controls after GBZ application (~ 450 spikes/sec) is similar to the peak firing rate for TC-exposed animals before GBZ application (~ 400 spikes/sec).

**Figure 8:**
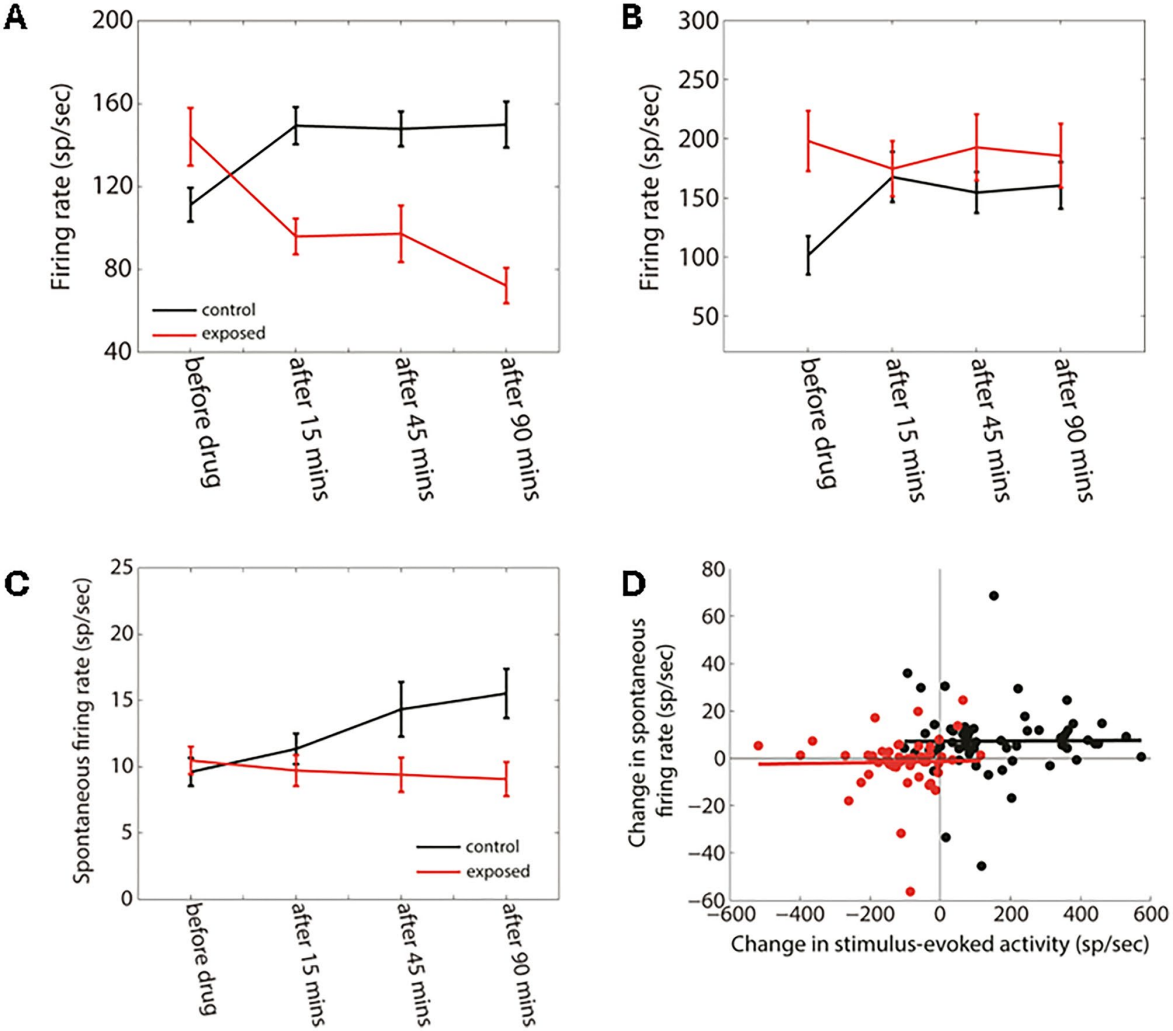
Averaged FR in the control (black line) and noise exposed (red line) groups as a function time for the two stimulus conditions (**A**, TC, **B**: STRF). Averaged SFR as a function time in control (black line) and noise-exposed (red line) groups (**C**). Changes in the SFR as a function of the changes in the stimulus-evoked activity (**D**). There is no correlation between the two variables (*p* > 0.05).

A linear mixed-effects analysis was used to evaluate the statistical changes in MUA (see methods) after GBZ application over time and group (Fig. 8). The firing rate was derived from the 10–50 ms post stimulus onset time window (at BF and 70 dB). For TC (Fig. 8A), we found a significant effect of group (F = 26.83, *p* < 0.0001), time (F = 27.57, *p* < 0.0001), and a significant two-way interaction between group and time (F = 17.44, *p* < 0.0001). Holm corrected post-hoc comparisons indicated that the firing rate in controls was significantly increased at all measured time points after GBZ application compared to before (*p* < 0.01). On the contrary, the firing rate was significantly decreased in exposed animals at all time points after GBZ application compared to before (*p* < 0.01). For STRF (Fig. 8B), a significant effect of group was found (F = 18.05, *p* < 0.001) as well as a significant two-way interaction between time and group (F = 6.577, *p* = 0.037). Post-hoc comparisons showed that the changes in firing rate after GBZ application compared to before was significant only at 15 min in both groups (control: *p* = 0.012; exposed: *p* = 0.03).

Finally, we investigated the effect of GBZ on the SFR in the two groups using a linear mixed-effects analysis (Fig. 8). There was no significant effect of group (F = 1.13, *p* = 0.28), time (F = 0.38, *p* = 0.82) or interaction between group and time (F = 2.05, *p* = 0.35). Moreover, we wanted to investigate if any changes in SFR could be correlated with changes in evoked pure tone activity (at BF and 70 dB). Changes in SFR were not significantly correlated with changes in evoked activity in control ( ${R}^{2}$= 0.000067, *p* = 0.951) and exposed (${R}^{2}$= 0.0004, *p* = 0.884) groups.

## Discussion

The main goal of the study was to provide further insights into the mechanisms of GABAergic homeostatic plasticity that may play a role in the well-documented neural hyperactivity in the auditory centers after noise exposure-induced hearing loss. First, we investigated whether the expression of KCC2 and NKCC1 in IC neurons, which are responsible for maintaining chloride homeostasis, is altered after noise-induced hearing loss. We show that KCC2 is dramatically downregulated in the DCN, VCN and IC 3 days after the noise trauma, and that the downregulation remains in DCN and IC at least until 30 days after the trauma. We did not find any expression of NKCC1 in these central regions in the control and noise-exposed groups. Second, we tested the effects of noise trauma on the action of a GABA antagonist (GBZ) on neuronal activity in IC. As expected, local administration of GBZ in the IC of controls increased the stimulus-evoked activity. On the other hand, local administration of GBZ in the IC of noise exposed animals decreased the stimulus-evoked activity.

The IC receives prominent GABAergic inputs mostly from the contralateral IC and superior olivary complex. The IC has also an extensive population of GABAergic neurons^32–35^. A considerable amount of evidence suggests that GABA mediates inhibition in the IC^36^ and this plays an important role in mediating stimulus-evoked non-monotonicity^31^, tonic inhibition^37^, shaping the frequency tuning in the IC^38^ (Fuzessery & Hall, 1996) and neuronal gain^39^.

It has long been assumed that central inhibition plays a strong role in the central hyperactivity that is systematically associated with hearing loss, including aging and noise-induced hearing loss^4,22,24,40,41^. Many studies have been devoted to investigating the effects of hearing loss on GABAergic neurotransmission in IC. In most studies, hearing loss is produced by noise trauma, but it can be induced by other causes such as, among others, cochleoctomy, aging or mechanical lesion of the cochlea. In IC, hearing loss has been found to result in a downregulation of GABA receptor (subunit alpha), glutamic acid decarboxylase (enzyme catalysing the conversion of glutamate to GABA), gene expression (including GABA receptor subunit alpha) and a decrease in the amplitude of evoked inhibitory synaptic currents^23,24,27,40,42–44^. In summary, most studies report that hearing loss is associated with a reduction of GABAergic neurotransmission in IC, suggesting that this reduction plays a role in the central hyperactivity that is triggered by hearing loss.

GBZ application in control animals is associated to a strong and expected increase in neural activity, broadly consistent with earlier studies and the well-known excitatory effect of GABA antagonist in IC^31,45,46^. The effect of GBZ in control animals depends on the characteristics of the rate-level function. For the neurons with non-monotonic function, GBZ increases the slope of the function dramatically, making the function monotonic. Hence, the difference between the averaged firing rate after and before GBZ application is −20 spikes/s at 40 dB and 80 spikes/s at 70 dB. For monotonic neurons, GBZ application simply shifts their rate-level function by about 20–30 spikes/s at all tested levels. These results suggest that GBZ has a multiplicative effect on non-monotonic neurons and an additive effect on monotonic neurons. They corroborate earlier results showing that GABAergic inputs are intensity-dependent in non-monotonic neurons, while they are intensity independent in monotonic neurons^31^. In the exposed group, on the other hand, the effect of GBZ is the exact opposite of that reported in controls. First, GBZ is associated to a strong reduction of stimulus-evoked activity. Second, the slope of the firing rate-level function is unchanged in non-monotonic neurons, while it is reduced in monotonic neurons. In other words, the effect of GBZ is uniform (40–60 spikes/s) for all levels in non-monotonic neurons, while it is larger at 70 dB in monotonic neurons (40 spikes/s). Overall, these results emphasize the strong plasticity of the GABAergic inhibition on the rate-level function.

Importantly, the effect of GABA on membrane potential largely depends on the intracellular concentration of chloride ions, which is itself regulated by KCC2 in adults^47^. In adult (control) animals, KCC2 maintains a low intracellular chloride concentration (relative to the extracellular chloride concentration) by extruding chloride ions from the neurons. Normal functioning of KCC2 in adult brains leads to the equilibrium potential for Cl- at the level below (more hyperpolarized) the neuronal resting membrane potential. As a consequence, the action of GABA is hyperpolarizing since GABA channel opening allows chloride to enter the cell. This is possible providing the fact that the resting membrane potential of neurons (in IC, the neuron’s resting membrane potential is around − 53 mV)^48^ is smaller than the equilibrium potential for chloride (~ − 65 mV). Interestingly, KCC2 has been found to be downregulated in several sensory systems after lesion, including the auditory system after unilateral cochlear nerve transection^25,26,28,29,49,50^. Our results are consistent with those of latter studies and extend them reporting that KCC2 can be downregulated after only partial hearing loss (affecting only high frequencies). Our study suggests that KCC2 regulation is part of the immense repertoire of mechanisms that play a role in the preservation of averaged neural activity roughly constant after hearing loss. It would be interesting to further investigate the relationship between KCC2 downregulation and hearing loss severity. It is conceivable that only modest effects on the cochlea, such as those limited to the synapses between inner hair cells and low-spontaneous cochlear fibers (i.e. “hidden hearing loss”)^51^, can also alter KCC2 function.

We observed that the KCC2 is downregulated up to 30 days after the noise trauma in the DCN and IC, while it is restored and even slightly upregulated in the VCN. This result suggests that the spatio-temporal sequence of KCC2 regulation after noise trauma can be complex. At this stage, it is unclear why the regulation of KCC2 is opposite between VCN and, DCN and IC, at 30 days after trauma. This may result from different inflammatory responses in these nuclei, since KCC2 downregulation depends on the release of pro-inflammatory mediators (microglial-derived BDNF)^52,53^. To note, the presence of inflammation has been reported in cochlear nucleus after hearing loss^28,54,55^. Alternatively, KCC2 regulation after noise trauma could reflect the post-translational consequences of kinase-mediated phosphoregulatory mechanisms, which have been shown to play a role in the plasticity of the GABAergic inhibition^21,56^.

*Importantly, our study does not provide a clear causal link between KCC2 downregulation and the effects of noise trauma on neural activity, and the effects of GBZ on neural activity*. We did not address whether KCC2 was downregulated in all neurons, or specifically in the GABA neurons or excitatory neurons. This result can have a strong impact on the global network activity. Indeed, KCC2 downregulation limited to GABA neurons may have a strong inhibitory effect on the global network activity, while it would be the opposite for KCC2 downregulation limited to excitatory neurons. Moreover, the balance between the excitatory and inhibitory networks could be altered by changes in the expression of glutamatergic, glycinergic or GABA receptors^4,20,40,57,58^. Further studies will be needed to address these questions. However, since our averaged data in the exposed group show that GBZ application is strongly and significantly inhibitory, this suggests that the net and macroscopic effect of GABA action on neural network activity is excitatory after noise trauma. On the other hand, the net (microscopic) effect of GABA and GBZ on membrane potential in individual neurons is unclear. Any robust demonstration of this effect, i.e. inhibitory-to-excitatory GABA-polarity switch, for example, would require intracellular characterization^52,59,60^. It is interesting to note here that evoked inhibitory post-synaptic currents in IC have been found to be depolarized from roughly − 64 to 40 mV, i.e. corresponding to a 24 mV depolarizing shift in inhibitory post-synaptic current equilibrium potential) after bilateral cochleoctomy^44^. This value is very close to what has been reported in the spinal cord after sciatic nerve injury (23.6 mV, from roughly − 73 mV to 49 mV)^26^. In the latter study, the downegulation of KCC2 co-transporters after the lesion has been linked to an inhibitory-to-excitatory GABA-polarity switch. It would be interesting if these studies could be carried out in the future in IC after noise trauma using whole-cell patch recordings.

Finally, while the effect of GBZ on stimulus-evoked activity is clear and strong, it is not statistically significant on the SFR. This differential effect could result from the fact that phasic and tonic inhibitions are not underpinned by the same mechanisms. Indeed, whereas phasic inhibition is mediated by synaptic GABA_A_ receptors, tonic inhibition is associated with extra-synaptic receptors and low concentration of ambient GABA^61^. Synaptic and extrasynaptic GABA_A_ receptors differ in their subunit compositions^62^. Assuming that SFR is mediated by tonic inhibition, the relative insensitivity of extrasynaptic GABA_A_ receptors to GBZ could explain the small, if any, effect of GBZ on SFR. However, according to the literature, it is possible that both synaptic and extrasynaptic GABAA receptors were blocked in IC at the concentration we used (10 µM)^63,64^. In this context, it would be very interesting to study the effects of extrasynaptic GABA_A_ receptor antagonist^65,66^ on SFR in the control and exposed groups. Indeed, a putative reduction of SFR in the exposed group by extrasynaptic GABA_A_ receptor antagonist could have important clinical implications (see the next paragraph).

It has been proposed that KCC2 downregulation after sensory lesion or nerve injury is adaptive. Indeed, reverting locally the central nervous system to an immature state, where KCC2 is downregulated and GABA excitatory^25^, may allow the central nervous system to adapt and reorganize (unmasking inputs, sprouting) to the sensory lesion or trauma^67^. However, this potential adaptive mechanism may come at a price: downregulation of KCC2 may also play a role in several pathophysiological conditions, such as epilepsy, neuropathic pain, spasticity, and neuropsychiatric disorders (schizophrenia and autism spectrum disorder)^21,26,53,56^. The mechanisms causing neuropathic pain has been well documented and is partially understood^68–70^. Sciatic nerve injury is causing ATP-mediated microglia activation (after activation of P2X receptor). As a result, the activated microglia can secret Brain-Derived Neurotrophic Factor (BDNF), which then binds to the TrkB receptor. Ultimately, this sequence results in a downregulation of KCC2, an accumulation of chloride ion into the cells and a change in the polarity of GABA action on membrane potential from inhibitory to excitatory^26,52^. Interestingly, blocking BDNF release from microglia prevents the effects of nerve injury on the anion reversal potential and pain (measured behaviorally in rats). These results are of considerable importance in the perspective of developing clinical therapies to manage neuropathic pain, and potentially other diseases related to neuronal hyperactivity after sensory lesion^21^. Moreover, the downregulation of KCC2 implies that the pathophysiological conditions related to neuronal hyperactivity cannot be treated simply by enhancing the GABAergic neurotransmission (GABA, glutamic acid decarboxylase, GABA receptors etc.). On the other hand, therapeutic approaches that can restore low intracellular chloride concentration may improve clinical symptoms such as neuropathic pain^21,53^. In this context, KCC2 is a target of choice for treatment and is preferable over GABA agonist, such as benzodiazepines, because it is directly and uniquely responsible for intracellular chloride concentration^59^.

It is tempting to speculate that GABA is less inhibitory in the IC after moderately severe hearing loss due to KCC2 downregulation. The reduced inhibition in the auditory centers may have strong functional consequences for the processing of all aspects of acoustic signals, including temporal and spectral processing and spatial hearing^2^. However, there remains the possibility that GABA is still inhibitory in other nuclei of the central auditory system, and in particular in the VCN, where KCC2 expression is restored (and even enhanced) 30 days after the noise trauma.

This paragraph is intended to discuss the potential implications of our results and also to propose potential links between various results obtained in the literature and KCC2 regulation that have so far not been developed. First, our results may contribute to further understand the putative mechanisms of tinnitus and/or hyperacusis. In the auditory system, the neural hyperactivity triggered after noise trauma has been suggested to be the neural corelates of tinnitus and hyperacusis^18,71–74^. The present study suggests that KCC2 downregulation plays a strong role in the noise-induced neural hyperactivity, at least when hearing loss is severe enough. The hearing loss produced by the trauma (115 dB, 4 h) is minimal below 4 kHz and larger than 60 dB above 8 kHz. This hearing loss is moderately severe and is relatively frequent in human subjects^75^. For future studies, it would be interesting to investigate more closely the relationship between the severity of hearing loss and the KCC2 downregulation. Our results provide a potential explanation for the paradoxical effects of benzodiazepines on tinnitus^76^. Moreover, they also provide insights into the paradoxical effects of diuretic drugs (furosemide and bumetanide), which may trigger or reduce tinnitus^73,77^. Indeed, KCC2 (and other cation-chloride co-transporters) are well-known to play a role in volume regulation of the cells^67^. This interpretation is consistent with the fact that angiotensin-converting enzyme inhibitor (involved in body water regulation) can elicit tinnitus as a side effect^78^. It has been shown that KCC2 is regulated by 5-hydroxytriptamine (5-HT) type 2A receptors to serotonin^79^. This regulatory mechanism may account, at least in part, for the link between the serotoninergic system and tinnitus and/or hyperacusis^76,80^. Finally, KCC2 downregulation and chloride dysregulation offer new avenues of understanding the potential link between auditory symptoms (tinnitus and/or hyperacusis) and neurological and psychiatric diseases such as autism spectrum disorder^21,81^. Indeed, it has been suggested that autism spectrum disorder may be associated to chloride dysregulation^25^. Since KCC2 seems to play a strong role in neural hyperactivity after hearing loss, KCC2 enhancer may represent a promising pharmacotherapeutic target for treating tinnitus and/or hyperacusis.

## Methods

The care and use procedures for animals in this study were approved by the Animal Care Committee of Bouches du Rhones, France. The project (APAFIS#20116-2019031117574974 v3, including all experimental procedures), has been evaluated and approved by the ethics committee n°071 and by Le Ministère de L’Enseignement Supérieur de la Recherche et de l’Innovation. All methods have been carried out in accordance with relevant guidelines and regulations. All methods are reported in accordance with ARRIVE guidelines (https://arriveguidelines.org). The age of guinea pigs used for the experiments was around 3–4 months old (9 to 20 weeks).

### Noise trauma

To induce high frequency hearing loss, guinea pigs were exposed to noise trauma in a sound booth room. Similar to previous study (Parameshwarappa et al. 2022), noise trauma stimulus consisted of a continuous pure tone set at 8 kHz, presented at 115 dB SPL for 4 h. Animals were housed inside a single polycarbonate cage and covered with a stainless steel lid. Noise trauma was presented through a speaker positioned on top of the cage so both ears were exposed to the trauma. The guinea pigs were awake during the noise exposure.

### Auditory brainstem responses

The hearing threshold and noise-induced hearing loss were assessed using auditory brainstem responses (ABRs) under anesthesia (mixture of Ketamine—46 mg/kg, and Medetomidine—50 mg/kg) injected intraperitoneally. ABRs were measured before (all animals) and at + 3 days (only in animals used for immunochemistry) and at + 30 days (all exposed animals) after noise trauma. The ABR recordings were collected using needle electrodes positioned under the skin at the skull vertex (active electrode), behind left or right mastoid (reference electrode), and in the neck muscle (ground electrode). Signals were amplified 104 times, filtered between 300 and 3000 Hz (Grass ICP 511 amplifiers), digitally converted, and averaged with a Micro1401 Plus system (Cambridge Electronic Devices, UK). Auditory stimuli were tone pips (with 2 ms linear rise/fall time and no plateau) at octave frequencies from 2 to 32 kHz at a repetition rate of 10 s^−1^ presented via an in-ear miniature earphone placed in one ear at a time. Tone pip level was varied from 90 dB SPL to 0 dB SPL using 10-dB steps. For each ear, stimulus repetitions incremented from 100 at 90 dB SPL to 1000 at 0 dB SPL. ABRs were analyzed offline using a custom written MATLAB program. The five ABR wave maxima corresponds to the different brainstem nuclei along the auditory pathway starting from the auditory nerve (wave I) to the inferior colliculus (wave V). The threshold was estimated as the lowest intensity of stimulation that yielded a repetitive waveform. Noise-induced hearing loss was evaluated by subtracting the ABR threshold obtained before and after the noise trauma.

### Immunochemistry

Immunochemical labelling of KCC2 and NKCC1 was performed according to previously validated protocols (Boulenguez et al. 2010; Tighilet et al. 2016). Twelve Hartley adult male guinea pigs (weighing between 400 and 800 g) were divided into 3 groups (n = 4). The first of the 3 groups was not subject to any noise trauma (control group). The other two groups were subjected to a 4-h 115 dB white noise trauma and sacrificed at 3 or 30 days post-trauma. Hearing loss was assessed by ABR before sacrifice in all cases.

For sacrifice, guinea pigs were deeply anaesthetised (mixture of Ketamine—46 mg/kg, and Medetomidine—50 mg/kg) injected intraperitoneally, before being killed by perfusion with 0.9% NaCl followed by 0.1 M podium phosphate, 4% paraformaldehyde pH 7.4 (1 ml/kg weight in both cases). The brain was extracted and preserved in perfusion solution for 24 h followed by subsequent 24 h baths in 0.1 M phosphate buffer at pH 7.4 with 10, 20 and 30% (w/v) of D-saccharose, respectively. The organ was then flash frozen in crushed dry ice and stored at − 80 °C.

Guinea pig brains were mounted and sliced in the coronal plane in a cryostat (Leica) for immunohistochemistry. The 30 μM sections containing the ventral cochlear nuclei (VCN), dorsal cochlear nuclei (DCN), and the central inferior colliculus (IC) were mounted onto SuperFrost®Plus slides (ThermoFisher Scientific). The location of the auditory brain structures was identified using guinea pig stereotaxis atlases. The mounted slides were then stored at -80 °C before use in immunochemistry.

Immunohistochemistry experiments were performed simultaneously on one animal from each experimental condition, resulting in 4 sets for these experiments. Slides were thawed and blocked at room temperature for 1 h in 0.1 M PBS containing 5% BSA. Slides were then immunochemically labelled by incubating at 4 °C overnight in the same solution containing primary antibody. The next day the slides were washed twice with 5 ml of 0.1 M PBS and incubated overnight at 4 °C with the appropriate Alexa fluor conjugated secondary antibody diluted in 0.1 M PBS. Slides were washed twice with 5 ml of 0.1 M PBS, stained with 4',6-Diamidino-2-Phenylindole, Dihydrochloride (DAPI) (Thermo Fisher Scientific) and mounted with coverslips in Roti®-Mount FluorCare (Carlroth). Control experiments consisting in omitting successively one of the primary antibodies were performed and resulted in the absence of cross-reactivity. All slides were stores at 4 °C before imaging. Imaging was performed using confocal microscopy on a Zeiss LSM 710 META laser-scanning microscope equipped with a 20 × lens and 2 × digital zoom. Each set was collected in approximately one month time with limited other users on the machine.

The anterior vs posterior positioning of each structure in our serial brain sections was used to confirm the identity of the structure in cresyl stained slices by cross-examining images against guinea pig stereotaxis atlases. After immunostaining, the structures were then identified in individual slices by examining each cut first with a 10× magnification on the confocal microscope and comparing it to the analogous cresyl stained brain sections. Individual images were then centered on the brain structure of interest and magnification was increased to 63× allowing us to visualise the individual cells used for the analysis. Since we used 40 µM sections all structures analysed appeared on several consecutive slides, the first and last slide identified for each structure were not used in the analysis, ensuring that all images were indeed of the structure to be analysed. We also used DAPI coloration to insure we were at a plane with cells.

A custom program written in Matlab (The Mathworks,Inc.) was developed to analyze fluorescence at the plasma membrane of neurons^28^. The background or non-specific immunofluorescence, was assessed by calculating the average fluorescence in a visually selected area devoid of neurons or any other stained structures. From this region, we then derived a threshold equal to the average immunofluorescence plus three times the standard deviation. All data were then subtracted from this threshold value and only positive values were conserved for further analysis. A region of interest was then drawn around the neuronal plasma membrane of each cell body. The program calculated the average fluorescence within the region of interest over data that were 20% above the maximum values. This thresholding insured that all pixels taken for calculating the average was part of the plasma membrane and that the same criterion was used for all slices in all conditions (otherwise, the averaged fluorescence can depend on how large the region of interest was manually drawn around the plasma membrane). Also, to avoid any biases at the stage of extracting the averaged fluorescence level in each condition, the experimenter in charge of the analysis (A.K.) ignored the conditions corresponding to the slices.

### Statistics

Putative differences for immunofluorescence between central regions (VCN, DCN and IC) and time (control, + 3 and + 30 days after trauma) have been tested with an analysis of variance, i.e. interaction between the central region and time. The Tuckey’s HSD (honestly significant difference) test was then used to performed post-hoc comparisons between different times for each central region. Statistical analysis has been done using R Stats Package (version 4.2.1).

### Electrophysiology

#### Animal preparation

Sixteen guinea pigs weighing between 400 and 800 g were divided into two groups: the control group (n = 8) and the noise exposed group (n = 8). Experiments in both groups were performed under anaesthesia. Anaesthesia was induced with a mixture of Ketamine (46 mg/kg) and Medetomidine (50 mg/kg) injected intraperitoneally. To maintain the effect of anaesthesia throughout the experiment, a half dose of Ketamine and Medetomidine were administrated every hour. Surgery was performed as described previously (Catz and Noreña 2013; Montejo and Noreña 2015; Parameshwarappa et al. 2022). Briefly, once the animals were anesthetized, an incision was made along the midline of the skull. The tissue overlying the frontal lobe was cleaned with a scalpel blade to remove any remaining tissues and fascia. Once cleaned, four small screws were fixed to the top of the skull and a large screw was fixed in between the small screws with the help of dental cement. The purpose of fixing the large screw was to provide a mechanically stable point of attachment to a metal head post. The tissue, skull, and dura overlying the inferior colliculus (12 mm caudal from bregma and 2.5 mm lateral from lambda) were removed over a 1 cm diameter, roughly. Throughout the entire experiment, the body temperature was maintained at around 37 °C with the thermostatically controlled heating blanket and rectal probe. After the experiment, a lethal dose of T61 was administrated.

### Acoustic stimulation

Stimuli were generated in MATLAB and transferred to an RP2.1- based sound delivery system (Tucker Davis Technologies) (Catz and Noreña 2013; Montejo and Noreña 2015). Acoustic stimuli were presented in a sound booth room from a headphone (Sennheiser HD595) placed 10 cm in front of the ear contralateral to the cortex where the recordings were carried out. The amplitude of each tone pip was adjusted to the transfer function of the sound delivery system to present the desired level in decibel sound pressure level (dB SPL).

The Tuning curve was determined using gamma tone pips (Montejo, 2015) with 49 tone pips covering 8 octaves (with 1/8 octave step), from 500 to 32,000 Hz, repeated 10 times. Tone pips were presented at different levels, from 70 to 30 dB SPL, with 10 dB step. Spontaneous firing rate (SFR) was recorded for 180 s of silence.

Spectro-Temporal Receptive Fields (STRFs) were obtained from 180-s multitone pip stimuli presented at 70 dB SPL (Catz and Noreña 2013; Montejo and Noreña 2015; Noreña et al. 2008). Tone pips consisting of 49 frequencies: 8 frequencies per octave covering 6 octaves between 500 and 32 kHz were presented randomly over time (independent Poisson process for each frequency with a rate of 2 Hz and a 50-ms dead time designed to prevent tones of the same frequency from overlapping in time). Tone pips of different frequencies could overlap in time. The envelope of the tone pips is given by y(t) = 〖(t/4)〗^2 e^(-t/4) with t in milliseconds (the stimulus duration is 50 ms, and the maximum amplitude is reached at 8 ms). The average rate of tone pip presentation was around 16 Hz/octave (considering the number of tone frequencies present per octave, along with the average presentation rate of each). Finally, SFR was recorded for 180 s of silence.

Electrophysiological recordings were all performed in the right central nucleus of inferior colliculus (IC) using linear probes of 16 electrodes spaced by 100 µm (model: A1 × 16^−10^ mm-100–177-CM16LP; NeuroNexus Technologies, Ann Arbor, MI). The electrode probe was manually advanced perpendicularly to the surface of the visual cortical up to a depth of 6 mm using a motorized micro-drive. Electrophysiological signals were acquired with Tucker-Davis System 3 Pentusa (TDT, Alachua, FL). The signals were then amplified 10,000 times with filter cut-off frequencies set at 2 Hz and 5 kHz. The amplified signals were processed by a TDT-System 3 Medusa multichannel data acquisition system. Multi-Unit Activity (MUA) was sampled at 24,414.5 Hz and was extracted from the 300 Hz high pass filtered signal.

As an initial step, to correctly localize the IC, a search procedure was used that consisted of recording MU and LFP activity induced by clicks, noise bursts, and tone pips (from 500 Hz to 32,000 Hz, 1/8 octave step). The IC was localized based on the presence of robust, short-latency response to tones (10–14 ms).

### Drug delivery

Based on several in-vivo studies, we used a 10 µM gabazine (GBZ) concentration since this concentration does not trigger any epileptiform neuronal activity (Darbin et al. 2006; Gaucher et al. 2013; Wang et al. 2009). A small quantity of the drug (0.1 ml) was delicately applied in the immediate vicinity of the electrode using a syringe. The drug was allowed to diffuse along the electrode shaft for about 4 min. Then, the cortical surface overlying the electrode (primary visual cortex) was rinsed with saline solution to remove any remaining residue of the drug. The visual inspection of neural activity did not show any epileptic activity in the IC after GBZ application. Moreover, we could observe that neural responses started to change 6 to 8 min after GBZ application. The effect of gabazine in both groups was studied at three different time points after the drug application (15 min, 45 min, and 90 min). The stability of neural activity before GBZ application was assessed (supplementary Fig. 1).

### Data analysis

All data were analysed using custom-written MATLAB routines. MUA or “spike-events” were detected by using an amplitude threshold on the high pass filtered data. The median was calculated on the negative values of the filtered signal; the threshold was then set to six times the median (Quiroga, 2006).

To generate tuning curves, the peak number of action potentials in the peri-stimulus time histogram (PSTH, 1-ms bin) were calculated over the first 100 ms after gamma-tone presentation. This was calculated per stimulus intensity and were combined into an intensity-frequency rate profile. The characteristic frequency was defined as the frequency that elicited a response at the lowest sound level.

The method for computing STRFs was similar to that used in previous studies (Catz and Noreña 2013; Montejo and Noreña 2015). STRFs for MUA were determined by constructing PSTHs, with time bins of 1 ms for each tone pip frequency. That is, spikes falling in the averaging time window (starting at the stimulus onset and lasting 100 ms) are counted. Because the average interstimulus interval in the stimulus ensemble (~ 10 ms) is smaller than the averaging time window, a spike can be counted in the PSTH of several pip frequencies. The frequency profiles were obtained by taking the average MUA response within a time window of 10–25 ms after stimulus onset and over all the frequencies. For averaging across all recordings, the frequency profiles were aligned according to their best frequency (BF) and then averaged (frequency was then expressed as octave difference from BF). The BF was the frequency at which the firing rate is maximum regardless of the level.

### Statistics

#### Tuning curve and STRF

Statistically significant changes in intensity-frequency-rate profile and STRF in control and noise exposed groups were tested using a non-parametric cluster analysis (Maris and Oostenveld 2007; Self et al. 2013). In the control and exposed animals, the t-scores (one sample t-tests) were calculated for the difference in intensity-frequency-rate profile (After GBZ application—Before) for each frequency-intensity pair, thus producing a 2-dimensional (2D) array of t-scores. For STRF, 2D t-maps was constructed for the difference in STRF maps (After GBZ application—Before) for each time–frequency pair. The 2D t-maps were then thresholded (all the non-significant values were set to zero). From the thresholded 2D array, adjacent t-scores with the same sign were clustered and a cluster statistic based on the sum of the absolute t-scores per cluster was calculated. To determine the significance of these clusters, the bootstrap method was used (cluster α threshold *p* < 0.05, two-tailed α level = 0.05). The data were randomly shuffled to generate random partitions. The intensity-frequency-rate profile (for STRF, time–frequency maps) before and after GBZ application were mixed with sampling with replacement. This procedure was repeated 1000 times to produce 1000 intensity-frequency- rate profiles. On each of those intensity-frequency- rate profile differences, the same procedure was done as applied to the real data, namely a t-score map was calculated, thresholded and a cluster analysis was performed. The maximum of the summed cluster value was taken as the bootstrap statistics. Hence, a distribution of test statistics was constructed. To evaluate significant clusters of the actual data, we compared the absolute t-value of each cluster to the test statistic distribution derived from the random partitions. Any cluster with a t-value above the 95th percentile of the shuffled distribution was considered significant. The same procedure was repeated at all measured time points. The peri-stimulus time histograms were tested statistically with the same method.

### PSTH

Similar to above, the statistical significance of PSTH differences between controls and chronic noise (or before and after acute noise trauma) was also tested using a non-parametric cluster analysis. Briefly, the cluster-based permutation test was conducted as follows: (1) the t-scores (one-sample t-tests) were calculated for the difference in PSTH (After-Before) at each time point, thus producing a 1-dimensional (1D) array of t-scores. Similarly, 1D array of t-scores (two-sample t-test) was also derived at each time point in the chronic study. (2) The time points with t-scores larger than the threshold were clustered based on temporal adjacency. The threshold was determined by the t-score corresponding to the *p*-value of 0.05. (3) We repeated steps (1) and (2) on the data permuted for 1000 times if clusters were identified in step (2). The cluster-level statistics were calculated by taking the maximum of the t-score within a cluster. The p-value of each cluster was given by the distribution of statistics on the permuted data. (4) We selected the temporal clusters with *p*-value ≤ 0.05 ≤ 0.05.

### Stimulus-evoked activity and spontaneous activity (MUA and LFP)

A linear mixed model was used to evaluate the effects of noise trauma on stimulus-evoked activity and SFR (lme4, R Core Team, 2018) (Bates et al. 2015). Fixed effects were group (control vs exposed), time (before, 15 min, 45 min, and 90 min), sound level (40, 50, 60, 70 dB) and rate-level function (monotonic vs. non-monotonic). We also tested for interactions between factors. The random effects were intercepts for animals. Visual inspection of residual plots did not reveal any obvious deviations from homoscedasticity or normality. *P*-values were obtained by likelihood ratio tests, i.e. running an Anova on the fitted data obtained from the full model (with all factors) and the model without the factor of interest. Post-hoc tests were done using Holm’s sequential Bonferroni correction.

### Supplementary Information

Supplementary Figures.

## Data Availability

The datasets generated during and/or analysed during the current study are available from the corresponding author on reasonable request.

## References

[1] Calford MB (2002). Dynamic representational plasticity in sensory cortex. Neuroscience.

[2] Herrmann B, Butler BE (2021). Hearing loss and brain plasticity: The hyperactivity phenomenon. Brain Struct. Funct..

[3] Wang Y, Hirose K, Liberman MC (2002). Dynamics of noise-induced cellular injury and repair in the mouse cochlea. J. Assoc. Res. Otolaryngol.

[4] Caspary DM, Llano DA (2017). Auditory thalamic circuits and GABAA receptor function: Putative mechanisms in tinnitus pathology. Hear Res..

[5] Eggermont JJ (2017). Acquired hearing loss and brain plasticity. Hear Res..

[6] Hsiao C-J, Galazyuk AV (2023). Depolarization shift in the resting membrane potential of inferior colliculus neurons explains their hyperactivity induced by an acoustic trauma. Front. Neurosci..

[7] Noreña AJ (2011). An integrative model of tinnitus based on a central gain controlling neural sensitivity. Neurosci. Biobehav. Rev..

[8] Turrigiano G (2011). Too many cooks? Intrinsic and synaptic homeostatic mechanisms in cortical circuit refinement. Annu. Rev. Neurosci..

[9] Schaette R, Kempter R (2006). Development of tinnitus-related neuronal hyperactivity through homeostatic plasticity after hearing loss: A computational model. Eur. J. Neurosci..

[10] Bakay WMH, Anderson LA, Garcia-Lazaro JA, McAlpine D, Schaette R (2018). Hidden hearing loss selectively impairs neural adaptation to loud sound environments. Nat. Commun..

[11] Hesse LL (2016). Non-monotonic relation between noise exposure severity and neuronal hyperactivity in the auditory midbrain. Front. Neurol..

[12] Schwartz O, Simoncelli EP (2001). Natural signal statistics and sensory gain control. Nat. Neurosci..

[13] Willmore BDB, King AJ (2023). Adaptation in auditory processing. Physiol. Rev..

[14] Auerbach BD, Rodrigues PV, Salvi RJ (2014). Central gain control in tinnitus and hyperacusis. Front. Neurol..

[15] Cody PA, Tzounopoulos T (2022). Neuromodulatory mechanisms underlying contrast gain control in mouse auditory cortex. J. Neurosci..

[16] Henton A, Zhao Y, Tzounopoulos T (2023). A role for KCNQ channels on cell type-specific plasticity in mouse auditory cortex after peripheral damage. J. Neurosci..

[17] Masri S (2021). Chemogenetic activation of cortical parvalbumin-positive interneurons reverses noise-induced impairments in gap detection. J. Neurosci..

[18] Noreña AJ, Farley BJ (2013). Tinnitus-related neural activity: Theories of generation, propagation, and centralization. Hear. Res..

[19] Turrigiano GG (2017). The dialectic of Hebb and homeostasis. Philos. Trans. R. Soc. Lond. B Biol. Sci..

[20] Caspary DM, Ling L, Turner JG, Hughes LF (2008). Inhibitory neurotransmission, plasticity and aging in the mammalian central auditory system. J. Exp. Biol..

[21] Doyon N, Vinay L, Prescott SA, De Koninck Y (2016). Chloride regulation: A dynamic equilibrium crucial for synaptic inhibition. Neuron.

[22] Resnik J, Polley DB (2017). Fast-spiking GABA circuit dynamics in the auditory cortex predict recovery of sensory processing following peripheral nerve damage. Elife.

[23] Argence M (2006). Modulation of inhibitory and excitatory synaptic transmission in rat inferior colliculus after unilateral cochleectomy: An in situ and immunofluorescence study. Neuroscience.

[24] Dong S, Rodger J, Mulders WHAM, Robertson D (2010). Tonotopic changes in GABA receptor expression in guinea pig inferior colliculus after partial unilateral hearing loss. Brain Res..

[25] Ben-Ari Y (2014). The GABA excitatory/inhibitory developmental sequence: A personal journey. Neuroscience.

[26] Coull JAM (2003). Trans-synaptic shift in anion gradient in spinal lamina I neurons as a mechanism of neuropathic pain. Nature.

[27] Vale C, Schoorlemmer J, Sanes DH (2003). Deafness disrupts chloride transporter function and inhibitory synaptic transmission. J. Neurosci..

[28] Tighilet B, Dutheil S, Siponen MI, Noreña AJ (2016). Reactive neurogenesis and down-regulation of the potassium-chloride cotransporter KCC2 in the cochlear nuclei after cochlear deafferentation. Front. Pharmacol..

[29] De Koninck Y (2007). Altered chloride homeostasis in neurological disorders: A new target. Curr. Opin Pharmacol..

[30] Parameshwarappa V, Pezard L, Noreña AJ (2022). Changes in the spatiotemporal pattern of spontaneous activity across a cortical column after noise trauma. J. Neurophysiol..

[31] Sivaramakrishnan S (2004). GABA(A) synapses shape neuronal responses to sound intensity in the inferior colliculus. J. Neurosci..

[32] Buentello DC, Bishop DC, Oliver DL (2015). Differential distribution of GABA and glycine terminals in inferior colliculus of rat and mouse. J. Comp. Neurol..

[33] Caspary DM, Raza A, Armour BL, Pippin J, Arneric SP (1990). Immunocytochemical and neurochemical evidence for age-related loss of GABA in the inferior colliculus: Implications for neural presbycusis. J. Neurosci..

[34] González-Hernández T, Mantolán-Sarmiento B, González-González B, Pérez-González H (1996). Sources of GABAergic input to the inferior colliculus of the rat. J. Comp. Neurol..

[35] Schofield BR, Beebe NL (2019). Subtypes of GABAergic cells in the inferior colliculus. Hear. Res..

[36] Faingold CL, Gehlbach G, Caspary DM (1989). On the role of GABA as an inhibitory neurotransmitter in inferior colliculus neurons: Iontophoretic studies. Brain Res..

[37] Lu Y, Jen PH-S, Zheng Q-Y (1997). GABAergic disinhibition changes the recovery cycle of bat inferior collicular neurons. J. Comp. Physiol. A.

[38] Fuzessery ZM, Hall JC (1996). Role of GABA in shaping frequency tuning and creating FM sweep selectivity in the inferior colliculus. J. Neurophysiol..

[39] Cai R, Kalappa BI, Brozoski TJ, Ling LL, Caspary DM (2014). Is GABA neurotransmission enhanced in auditory thalamus relative to inferior colliculus?. J. Neurophysiol..

[40] Dong S, Mulders WHAM, Rodger J, Woo S, Robertson D (2010). Acoustic trauma evokes hyperactivity and changes in gene expression in guinea-pig auditory brainstem: Brainstem plasticity after acoustic trauma. Eur. J. Neurosci..

[41] Richardson BD, Sottile SY, Caspary DM (2021). Mechanisms of GABAergic and cholinergic neurotransmission in auditory thalamus: Impact of aging. Hear. Res..

[42] Abbott SD, Hughes LF, Bauer CA, Salvi R, Caspary DM (1999). Detection of glutamate decarboxylase isoforms in rat inferior colliculus following acoustic exposure. Neuroscience.

[43] Mossop JE, Wilson MJ, Caspary DM, Moore DR (2000). Down-regulation of inhibition following unilateral deafening. Hear. Res.

[44] Vale C, Sanes DH (2000). Afferent regulation of inhibitory synaptic transmission in the developing auditory midbrain. J. Neurosci..

[45] Pollak GD, Park TJ (1993). The effects of GABAergic inhibition on monaural response properties of neurons in the mustache bat’s inferior colliculus. Hear. Res..

[46] Gourévitch B, Mahrt EJ, Bakay W, Elde C, Portfors CV (2020). GABAA receptors contribute more to rate than temporal coding in the IC of awake mice. J. Neurophysiol..

[47] Chamma I, Chevy Q, Poncer JC, Levi S (2012). Role of the neuronal K-Cl co-transporter KCC2 in inhibitory and excitatory neurotransmission. Front. Cell. Neurosci..

[48] Pedemonte M, Torterolo P, Velluti RA (1997). In vivo intracellular characteristics of inferior colliculus neurons in guinea pigs. Brain Res..

[49] Boulenguez P (2010). Down-regulation of the potassium-chloride cotransporter KCC2 contributes to spasticity after spinal cord injury. Nat. Med..

[50] Pozzi D, Rasile M, Corradini I, Matteoli M (2020). Environmental regulation of the chloride transporter KCC2: Switching inflammation off to switch the GABA on?. Transl. Psychiatry.

[51] Liberman MC, Kujawa SG (2017). Cochlear synaptopathy in acquired sensorineural hearing loss: Manifestations and mechanisms. Hear. Res..

[52] Coull JAM (2005). BDNF from microglia causes the shift in neuronal anion gradient underlying neuropathic pain. Nature.

[53] Gagnon M (2013). Chloride extrusion enhancers as novel therapeutics for neurological diseases. Nat. Med..

[54] Baizer JS (2015). Effects of acoustic trauma on the auditory system of the rat: The role of microglia. Neuroscience.

[55] Fuentes-Santamaría V (2017). The role of glia in the peripheral and central auditory system following noise overexposure: Contribution of TNF-α and IL-1β to the pathogenesis of hearing loss. Front. Neuroanat..

[56] Kahle KT (2015). K-Cl cotransporters, cell volume homeostasis, and neurological disease. Trends Mol. Med..

[57] Balaram P, Hackett TA, Polley DB (2019). Synergistic transcriptional changes in AMPA and GABAA receptor genes support compensatory plasticity following unilateral hearing loss. Neuroscience.

[58] Milbrandt JC, Holder TM, Wilson MC, Salvi RJ, Caspary DM (2000). GAD levels and muscimol binding in rat inferior colliculus following acoustic trauma. Hear. Res..

[59] Mapplebeck JCS (2019). Chloride dysregulation through downregulation of KCC2 mediates neuropathic pain in both sexes. Cell Rep..

[60] Pressler RT, Strowbridge BW (2022). Extra-glomerular excitation of rat olfactory bulb mitral cells by depolarizing GABAergic synaptic input. J. Neurosci..

[61] Belelli D (2009). Extrasynaptic GABAA receptors: Form, pharmacology, and function. J. Neurosci..

[62] Wu X (2012). γ-Aminobutyric acid type A (GABAA) receptor α subunits play a direct role in synaptic versus extrasynaptic targeting. J. Biol. Chem..

[63] Stell BM, Mody I (2002). Receptors with different affinities mediate phasic and tonic GABA(A) conductances in hippocampal neurons. J. Neurosci..

[64] Ruiz A, Campanac E, Scott RS, Rusakov DA, Kullmann DM (2010). Presynaptic GABAA receptors enhance transmission and LTP induction at hippocampal mossy fiber synapses. Nat. Neurosci..

[65] Darmani G (2016). Effects of the selective α5-GABAAR antagonist S44819 on excitability in the human brain: A TMS-EMG and TMS-EEG phase I study. J. Neurosci..

[66] Etherington L-A (2017). Selective inhibition of extra-synaptic α5-GABAA receptors by S44819, a new therapeutic agent. Neuropharmacology.

[67] Payne JA, Rivera C, Voipio J, Kaila K (2003). Cation-chloride co-transporters in neuronal communication, development and trauma. Trends Neurosci..

[68] Costigan M, Scholz J, Woolf CJ (2009). Neuropathic pain: A maladaptive response of the nervous system to damage. Annu. Rev. Neurosci..

[69] Finnerup NB, Kuner R, Jensen TS (2021). Neuropathic pain: From mechanisms to treatment. Physiol. Rev..

[70] Woolf CJ, Salter MW (2000). Neuronal plasticity: Increasing the gain in pain. Science.

[71] Eggermont JJ, Roberts LE (2004). The neuroscience of tinnitus. Trends Neurosci..

[72] Mulders WHAM, Ding D, Salvi R, Robertson D (2011). Relationship between auditory thresholds, central spontaneous activity, and hair cell loss after acoustic trauma. J. Comp. Neurol..

[73] Mulders WHAM, McMahen C, Robertson D (2014). Effects of chronic furosemide on central neural hyperactivity and cochlear thresholds after cochlear trauma in Guinea pig. Front. Neurol..

[74] Longenecker RJ, Galazyuk AV (2016). Variable effects of acoustic trauma on behavioral and neural correlates of tinnitus in individual animals. Front. Behav. Neurosci..

[75] Goman AM, Lin FR (2016). Prevalence of hearing loss by severity in the United States. Am. J. Public Health.

[76] Langguth B, Elgoyhen AB, Cederroth CR (2019). Therapeutic approaches to the treatment of tinnitus. Annu. Rev. Pharmacol. Toxicol..

[77] Risey JA, Guth PS, Amedee RG (1995). Furosemide distinguishes central and peripheral tinnitus. Int. Tinnitus J..

[78] Elgoyhen AB (2014). Identifying tinnitus-related genes based on a side-effect network analysis. CPT Pharmacomet. Syst. Pharmacol..

[79] Bos R (2013). Activation of 5-HT2A receptors upregulates the function of the neuronal K-Cl cotransporter KCC2. Proc. Natl. Acad. Sci. U.S.A..

[80] Oishi N (2010). Effects of selective serotonin reuptake inhibitor on treating tinnitus in patients stratified for presence of depression or anxiety. Audiol. Neurootol..

[81] Williams ZJ, He JL, Cascio CJ, Woynaroski TG (2021). A review of decreased sound tolerance in autism: Definitions, phenomenology, and potential mechanisms. Neurosci. Biobehav. Rev..

