# Supplementary material for: Noise-induced hearing loss alters potassium-chloride cotransporter KCC2 and GABA inhibition in the auditory centers
Source: Sci Rep. 2024 May 9;14:10689. doi: 10.1038/s41598-024-60858-1 (PMC11082187; doi:10.1038/s41598-024-60858-1)
Supplement: Supplementary file 1 — Supplementary Figures. [file 41598_2024_60858_MOESM1_ESM.pdf]

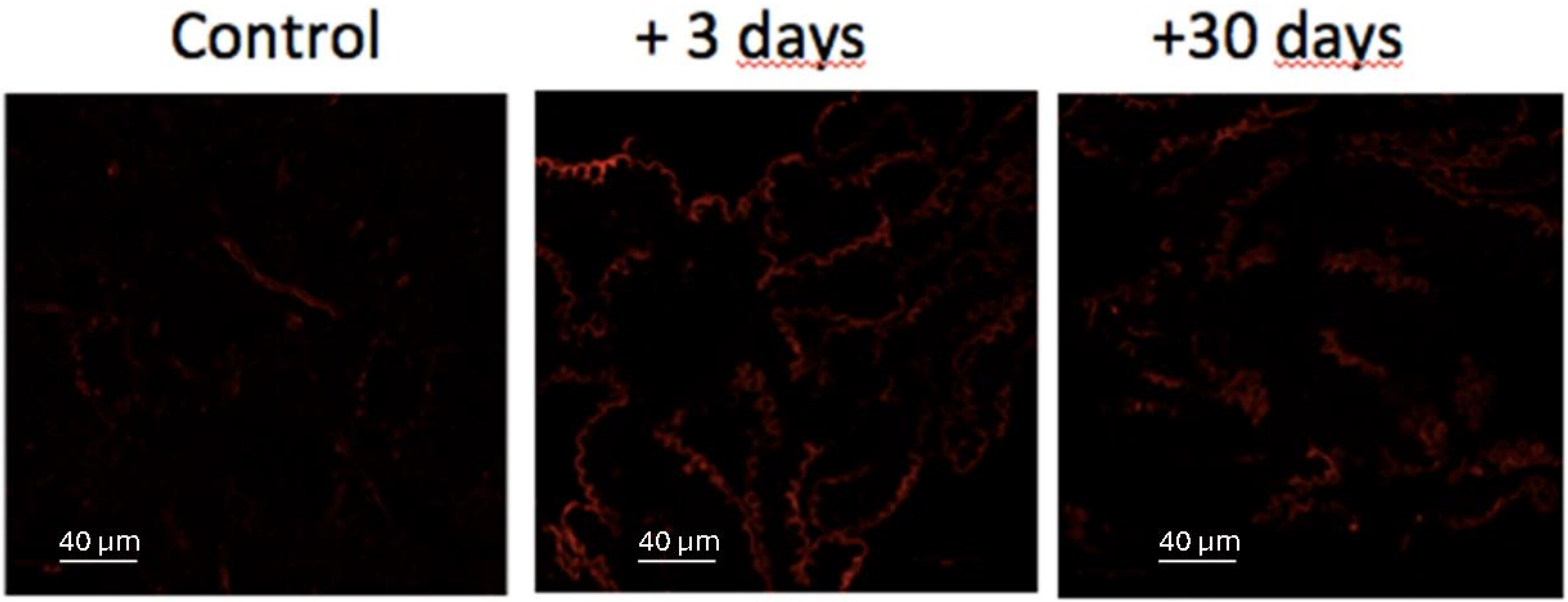

Supplementary figure 1: Examples of slices showing NKCC1-related fluorescence in control (first column) and exposed animals (second and third columns: +3d and +30d after noise trauma, respectively). These slices show the NKCC1-related fluorescence in the cochlear nerve, which is especially marked at 3 and 30 days after noise trauma. We did not quantify the NKCC1-related fluorescence in the cochlear nerve since it was not the purpose of this study to address this question. However, this fluorescence demonstrates that the antibodies used in this study to reveal membrane NKCC1 were functioning properly.

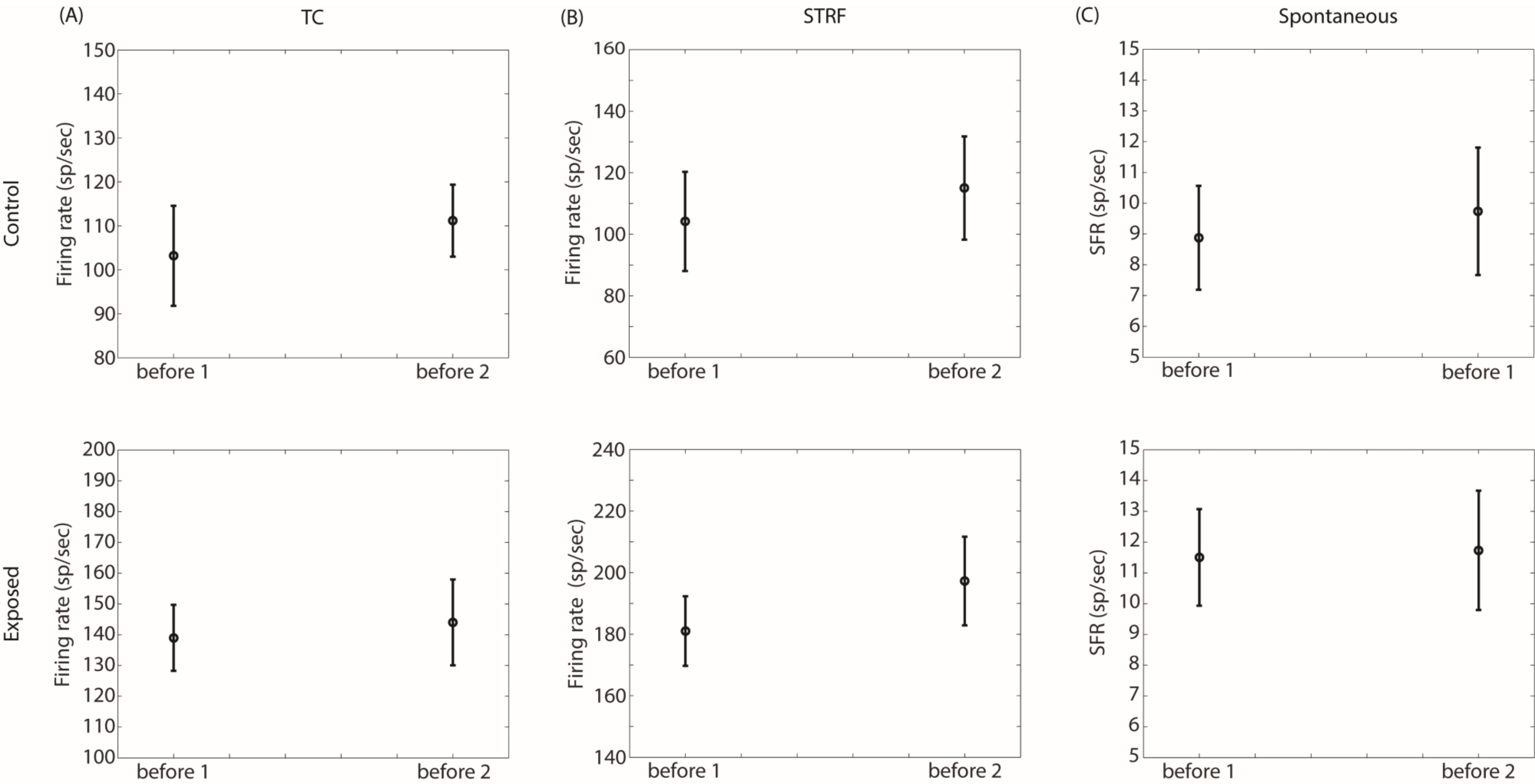

Supplementary figure 2: Multi-unit activity measured during stimulus-evoked (pure-tone and multi-tone pips) and SFR at two different time points before GBZ application. No significant difference in firing rate was observed between the two measured time points (Wilcoxon rank-sum test, $p > 0.05$).
